# Mitochondrial Respiratory Pathways Inhibition in *Rhizopus oryzae* Potentiates Activity of Posaconazole and Itraconazole via Apoptosis

**DOI:** 10.1371/journal.pone.0063393

**Published:** 2013-05-17

**Authors:** Fazal Shirazi, Dimitrios P. Kontoyiannis

**Affiliations:** Department of Infectious Diseases, Infection Control and Employee Health, Unit 402, University of Texas MD Anderson Cancer Center, Houston, Texas, United States of America; Geisel School of Medicine at Dartmouth, United States of America

## Abstract

The incidence of mucormycosis has increased drastically in immunocompromised patients. Also the array of targets whose inhibition results in Mucorales death is limited. Recently, researchers identified mitochondria as important regulators of detoxification and virulence mechanisms in fungi. In this context, targeting the mitochondrial respiratory chain may provide a new platform for antifungal development. We hypothesized that targeting respiratory pathways potentiates triazoles activity via apoptosis. We found that simultaneous administration of antimycin A (AA) and benzohydroxamate (BHAM), inhibitors of classical and alternative mitochondrial pathways respectively, resulted in potent activity of posaconazole (PCZ) and itraconazole (ICZ) against *Rhizopus oryzae*. We observed cellular changes characteristic of apoptosis in *R. oryzae* cells treated with PCZ or ICZ in combination with AA and BHAM. The fungicidal activity of this combination against *R. oryzae* was correlated with intracellular reactive oxygen species accumulation (ROS), phosphatidylserine externalization, mitochondrial membrane depolarization, and increased caspase like activity. DNA fragmentation and condensation assays also revealed apoptosis of *R. oryzae* cells. These apoptotic features were prevented by the addition of the ROS scavenger *N*-acetyl-cysteine. Taken together, these findings suggest that the use of PCZ or ICZ in combination with AA and BHAM makes *R. oryzae* exquisitely sensitive to treatment with triazoles via apoptosis. This strategy may serve as a new model for the development of improved or novel antifungal agents.

## Introduction

The mortality rate in immunocompromised patients with mucormycosis remains high [1], [2]. The genus *Rhizopus* was first described in 1821 by Ehrenberg and belongs to the order Mucorales in the phylum Zygomycota [3]. Approximately 60% of all disease manifestations and 90% of all rhinocerebral cases are caused by *Rhizopus oryzae* [3]. The rapid growth rate and the angioinvasive nature of the disease lead to an overall mortality of 50% [3]. *R. oryzae*, is resistant to most antifungal agents, with the exception of the polyene amphotericin B (AMB) and the third generation azole, posaconazole (PCZ) [2]. Both AMB/PCZ target the cell membrane, the need for identification of novel cellular targets whose inhibition results in loss of viability of Mucorales species is dire.

Recent reports revealed that mitochondria play a fundamental role in oxidative stress responses induced by antifungal drugs, as well as a prominent role in fungal pathogenesis [4]–[6]. In pathogenic fungi, the evolutionarily -conserved mitochondrial respiratory pathways function as important “circuits” for fungal homeostatic cell responses, which counteract the damaging effects of antifungals, including effects on the development and maintenance of antifungal resistance [7]. Specifically, the mitochondrial respiratory pathways are involved in ATP synthesis and play important roles in the generation of reactive oxygen species (ROS), calcium homeostasis, and apoptosis [6]. Therefore, the oxidative stress response pathways of fungal mitochondria appear to be promising targets for the antifungal agents [7].

Recent genomic analysis of *R. oryzae* revealed evidence of an ancestral whole- genome duplication event that expanded gene families related to cell wall synthesis, the ergosterol -biosynthesis encoding genes, and other key homeostatic cell responses compared to more common molds, such as *Aspergillus* species [3]. As a consequence, *R. oryzae* appears to be genetically well-equipped to resist the toxic effects of antifungals, including the ergosterol-depleting triazoles such as PCZ and itraconazole (ICZ) [8]. This genome duplication also resulted in the duplication of nearly all subunits of the protein complexes associated with respiratory electron transport chains, the ATPases, and the ubiquitin-proteasome systems. Retention of duplicated protein complexes involved in energy generation in *R. oryzae* provided a rapid growth advantage for this organism. Therefore, simultaneous targeting of ergosterol synthesis and mitochondrial pathways in *R. oryzae* may be an important strategy for enhancing the potency of triazoles against this devastating pathogen.

It is unclear whether mitochondrial inhibitors increase the activity of antifungal drugs. Antimycin A (AA), an inhibitor of complex III (classical pathway) and benzohydroxamate (BHAM), an inhibitor of cytochrome *b* (alternative pathway) were used to study the mitochondrial respiratory pathways in *Rhizopus stolonifer and Candida parapsilosis* [9], [10]. Using these respiratory inhibitors, respiratory complexes (alternative oxidase, external NADH dehydrogenase, and the glycerol phosphate shuttle, in addition to the classic complexes I, II, III, IV, and V) were described in *R. stolonifer.* The enhancement of caspofungin activity was also studied *in C. parapsilosis* in the presence of AA and BHAM [9], [10]. Thus, herein, we examined for the first time the effects of simultaneous inhibition of classical and alternate mitochondrial pathways by treatment with AA and BHAM, on PCZ and ICZ activity using *in vitro* methods to characterize the antifungal activity of these triazoles against *R. oryzae.* We found that AA and BHAM significantly enhanced the potency of PCZ and ICZ against *R. oryzae* in vitro and our results suggest this enhancement is mediated by apoptosis.

## Materials and Methods

### Drugs

PCZ (5 mg/ml; Merck & Co., Inc.) and fluconazole (FLC; 2 mg/ml; Pfizer) stocks were prepared in sterile distilled water. ICZ (5 mg/ml; Janssen Pharmaceuticals), AA (200 µM; Sigma), and BHAM (200 µM; Sigma) stocks were prepared in ethanol, and aliquots of them were stored at −20°C in the dark until use.

### *R. oryzae* Isolate and Growth Conditions

A clinical *R. oryzae* isolate (*R.o*-969) was allowed to grow on Sabouraud dextrose agar plates. After 48 h of incubation at 37°C, *R. oryzae* spores were collected and washed twice in sterile phosphate-buffered saline (PBS). The spores were then counted using a hemocytometer and stored at 4°C in PBS.

### Susceptibility Testing

Broth microdilution was performed according to the Clinical and Laboratory Standards Institute method [11]. Briefly, twofold serial drug (PCZ, ICZ, and FLC) dilutions were prepared in flat-bottomed microtiter plates (100 µl/well) in the presence or absence of AA and BHAM. Inhibitory concentrations of AA and BHAM were used in all experiments (1.5 nM and 1.5 µM, respectively). Drug-free wells were used as controls. Each well was inoculated with 100 µl of freshly isolated *R. oryzae* spores (2–3 days old; 1×10^4^ spores/ml), suspended in the test medium. After 48 h of incubation at 37°C, the MICs of PCZ, ICZ, and FLC were determined visually as the lowest drug concentrations resulting in complete growth inhibition. To determine the MFCs of PCZ, ICZ, and FLC, an aliquot (20 µl) taken from each well exhibiting 100% growth inhibition was plated onto YPD agar (1% yeast extract, 2% peptone, 2% dextrose, and 2% agar) plates. After 24 h of incubation at 37°C, the MFC was recorded as the lowest drug concentration, at which no growth was observed.

### Viability Assay

Apoptosis affects the ionic gradient across the plasma membrane, leading to ionic imbalance and depolarization of the membrane [12]. *R. oryzae* germlings were stained with bis-[1, 3-dibutylbarbituric acid] trimethine oxonol (DiBAC, Molecular Probes) as previously described [13]. Briefly, *R. oryzae* spores (10^6^/ml) were allowed to grow to germlings in microcentrifuge tubes in RPMI 1640 medium containing 0.15% (wt/vol) polyacrylic acid (Junlon; Nihon Junyaku) in all experiments to prevent hyphal aggregation at 37°C with shaking for 5 h. The medium was removed via centrifugation at 13,000×*g*, and germlings were resuspended in RPMI 1640 medium containing both AA and BHAM along with PCZ or ICZ (0.06–0.25 µg/ml) for 3 h at 37°C. After incubation, germlings were washed twice in 0.1 M 3-(N-morpholino) propanesulfonic acid (pH 7) to remove the drugs and incubated with 2 µg/ml DiBAC. After 1 h of incubation at room temperature (RT) in the dark, samples of *R. oryzae* germlings were washed twice in 3-(N-morpholino) propanesulfonic acid, and germlings were mounted on glass slides. Images were acquired using a fluorescence microscope (Nikon Microphot SA) with an FITC filter at a magnification of 400×. This experiment was also conducted in the presence of the ROS scavenger *N-*acetyl cysteine (NAC) at a concentration of 40 mM, at which the maximum number of *R. oryzae* germlings exhibited reversal of the antifungal effect of PCZ and ICZ via increased survival.

### Detection of Intracellular ROS Accumulation in *R. oryzae* Germlings

ROS play important roles as early initiators of apoptosis in yeast and other filamentous fungi [14]. Intracellular ROS levels in *R. oryzae* germlings were measured using a fluorimetric assay with dihydrorhodamine (DHR)-123 (Sigma) staining [14], [15]. The germlings were treated with 0.06–0.25 µg/ml PCZ or ICZ in combination with AA and BHAM for 3 h at 37°C. These germlings were spiked with DHR-123 (5 µg/ml) and incubated for an additional 2 h at RT. Germlings were then harvested and viewed directly under a fluorescence microscope equipped with a filter set with an excitation limit of 500 nm and emission limit of 550 nm. Alternatively, fluorescence intensity values of *R. oryzae* germlings stained with DHR-123 were determined using a POLARstar Galaxy microplate reader (BMG LABTECH) with an excitation limit of 500 nm and an emission limit of 550 nm. Fluorescence intensity was expressed as relative fluorescence unit, a unit of measurement used in analysis of fluorescence. The same experiment was conducted in the presence of NAC at a concentration of 40 mM.

### Measurement of the ΔΨ_m_ of *R. oryzae* Germlings

ΔΨ_m_, an indicator of the energetic state of mitochondria and cells, was used to assess the activity and depolarization of the mitochondrial permeability in *R. oryzae* germlings [16], [17]. To determine the effect of treatment with PCZ or ICZ in combination with AA and BHAM on the ΔΨ_m_ of *R. oryzae* germlings fluorescence was measured using rhodamine (Rh)-123 staining with fluorescence microscopy and a fluorescence polarization microplate reader according to a procedure described by Wu et al. [18]. Germlings exposed to PCZ or ICZ in combination with AA and BHAM at 37°C for 3 h were harvested via centrifugation, washed twice, and resuspended in PBS. Rh-123 was added to the final concentration (10 µM), and the mixture was incubated for 30 min in the dark at RT. ΔΨ_m_ was expressed as the fluorescence intensity of Rh-123, which was read at 488-nm excitation and 525-nm emission. The same experiment was conducted in the presence of NAC at a concentration of 40 mM.

### Annexin V/PI Double Staining of *R. oryzae* Cells

The apoptosis marker phosphatidylserine (PS) is located on the inner leaflet of the lipid bilayer of the cytoplasmic membrane and translocated to the outer leaflet at the onset of apoptosis [15]. It can be detected using staining with annexin V- fluorescein isothiocyanate (FITC), which binds to PS with high affinity in the presence of Ca^2+^ [19]. Briefly, the cell wall of *R. oryzae* germlings were digested with a lysing enzyme mixture (1 U of chitosanase, 1.3 U of chitinase, 1 U of lyticase, and 10 mg/ml lysing enzyme; Sigma) for 5 h at 30°C and pretreated with 0.060–0.125 µg/ml of PCZ or ICZ combined with AA and BHAM for 3 h at 37°C. The digested cells were then stained with annexin V-FITC (BD Pharmingen) and propidium iodide (PI) at RT for 15 min and observed under a fluorescence microscope to assess the externalization of PS as described by Madeo et al. [15]. This experiment was also conducted in the presence of NAC at a concentration of 40 mM.

### Measurement of DNA Damage in *R. oryzae* Germlings

DNA fragmentation, a key apoptotic phenotype [12] and a characteristic change in apoptosis, was detected in *R. oryzae* germlings using a terminal deoxynucelotidyltransferase-dUTP nick end-labeling (TUNEL) assay [18]. Specifically, *R. oryzae* germlings pretreated with 0.060–0.125 µg/ml PCZ or ICZ along with AA plus BHAM for 3 h at 37°C were fixed with 3.7% formaldehyde for 30 min on ice and digested with a lysing enzyme mixture. The germlings were then rinsed twice with PBS and incubated in a permeabilization solution (0.1% Triton X-100 and 0.1% sodium citrate) for 2 min on ice. Subsequently, germlings were rinsed twice with PBS and incubated with 50 µl of a DNA-labeling solution for 60 min at 37°C. After incubation, the germlings were rinsed three times with PBS and incubated with 100**µl of anti-BrdU fluorescein (Sigma) for 30 min at RT as per the manufacturer’s instructions. The cells were then observed for fluorescence with excitation and emission wavelengths of 488 nm and 520 nm, respectively.

Chromatin condensation and fragmentation are well-described cytological hallmarks of apoptosis. Chromatin condensation was assessed in *R. oryzae* germlings pretreated with 0.060–0.125 µg/ml PCZ or ICZ combined with AA and BHAM for 3 h at 37°C. After incubation, germlings were stained with 3 µg/ml 4′6-diamino-2-phenylindole (DAPI, Sigma) in PBS for 10 min at RT in the dark [20]. Germlings were then observed for fluorescence with excitation and emission wavelengths of 350 nm and 461 nm, respectively.

### Detection of Metacaspase (Caspase-like) Activity in *R. oryzae* Germlings

Metacaspases are caspase-like cysteine proteases identified in yeasts, plants, and protozoa [21]. Metacaspases are cysteine-dependent proteases found in protozoa, fungi and plants and are distantly related to metazoan caspases. Although metacaspases share structural properties with those of caspases, they lack aspartic acid (Asp) specificity and cleave their targets after Arg or Lys residues. A metacaspase-specific molecular probe for measuring and inhibiting metacaspase activity is not available [21], [22]. We detected caspase-like proteolytic activity in fungi by using FITC-VAD-FMK probe [20]. They are closely associated with generation of ROS and mitochondrial dysfunction [12]. Active caspase-like proteolytic activity was detected in *R. oryzae* germlings pretreated with 0.060–0.125 µg/ml PCZ or ICZ combined with AA and BHAM for 3 h at 37°C using the CaspACE FITC-VAD-FMK In Situ Marker (Promega) according to the manufacturer's instructions [20]. Cells with intracellularly active caspases stained fluorescent green, whereas nonapoptotic cells were unstained [20]. Pretreated *R. oryzae* germlings were washed in PBS, resuspended in 10 µM CaspACE FITC-VAD-FMK, and incubated for 2 h at 30°C. After incubation, germlings were washed twice and resuspended in PBS. The germlings were observed microscopically for fluorescence with excitation and emission settings of 488 nm and 520 nm, respectively.

### Superoxide Dismutase (SOD, EC 1.15.1.1) in *R. oryzae* Germlings

Excess ROS are scavenged by the antioxidant enzymes catalase, SOD, and glutathione peroxidase [23].The SOD activity in *R. oryzae* germlings pretreated with 0.060–0.125 µg/ml PCZ or ICZ along with AA and BHAM for 3 h at 37°C was measured as described by Arora et al. [24]. Briefly, 20 µl of 100 mM hydroxylamine hydrochloride and 100 µL of a cell extract were added to 30 µl of 1.6 mM nitroblue tetrazolium (NBT) and 6 µl of 10% Triton X-100, and the volume was made up to 2.1 ml with the addition of sodium carbonate buffer (50 mM, pH 10.2). The rate of NBT reduction was measured at 560 nm for 5 min using a spectrophotometer. One unit of SOD was expressed as the amount of protein required to inhibit the rate of reduction of NBT by 50%.

### Cytochrome *c* (cyt *c*) Release from Mitochondria

Translocation of cyt *c* from mitochondria to the cytosol is a critical event in apoptosis [12]. For estimation of cyt *c*, isolation of mitochondria from *R. oryzae* germlings was performed according to the method described by Niimi et al. [25]. Briefly, *R. oryzae* germlings were allowed to grow in RPMI broth at 37°C for 5 h. They were then resuspended in fresh RPMI broth and further incubated with 0.060–0.125 µg/ml PCZ or ICZ in the presence of both AA and BHAM at 37°C for 3 h. After centrifugation at 5,000×*g* for 5 min, the pellet was resuspended in a homogenization medium (50 mM Tris, pH 7.5, 2 mM ethylenediaminetetraacetic acid [EDTA], 1 mM phenylmethylsulfonyl fluoride) and homogenized. Next, the homogenization medium was supplemented with 2% glucose and centrifuged at 2,000×*g* for 10 min to remove cellular debris and unbroken cells. The supernatant was collected and centrifuged at 30,000×*g* for 45 min. The supernatant was used to estimate cyt *c* in cytoplasm and the pellet was resuspended in 50 mM Tris (pH 5.0) and 2 mM EDTA, incubated for 5 min, and centrifuged at 5,000×*g* for 30 s. The pellet was then collected for determination of cyt *c* remaining in mitochondria. Mitochondria were suspended in 2 mg/ml of Tris-EDTA buffer. After being reduced by 500 mg/ml ascorbic acid at RT for 5 min, the quantities of cyt *c* in the supernatants and mitochondria were determined by measuring the absorbance at 550 nm with a POLARstar Omega spectrophotometer (BMG LABTECH). Results were expressed as relative percentage, defined as percentage of cyt c in mitochondria or cytoplasm compared to controls (AA+BHAM).

#### Estimation of protein

Protein was estimated according to a method described by Lowry *et al*. [26], using crystalline bovine serum albumin as a standard.

#### Statistical analysis

For all assays, three independent experiments were carried out on 3 different days. Comparisons of multiple treatment groups were performed using two-way analysis of variance with post-hoc pairwise comparisons by Dunnett’s test. Calculations were performed using the InStat software program (GraphPad Software). Two tailed *P* values of less than 0.05 were considered to be statistically significant.

## Results

### When Combined with AA and BHAM, PCZ and ICZ Profoundly Inhibit *R. oryzae* Growth and Become Fungicidal

AA and BHAM had no effect on *R. oryzae* growth alone or in combination without the triazoles (Table 1). Similarly, use of either AA or BHAM in combination with PCZ or ICZ had no synergistic effects. However, the addition of both AA and BHAM to treatment with PCZ or ICZ resulted in a large (32-fold) decrease in minimum inhibitory concentrations (MICs) of the triazoles. Similarly, the minimum fungicidal concentrations (MFCs) were 4 µg/ml for PCZ and 8 µg/ml for ICZ in combination with AA and BHAM. In contrast, PCZ and ICZ alone had no fungicidal effects.

**Table 1:** Effects of AA and BHAM at inhibitory concentrations on the MICs of PCZ and ICZ against *R. oryzae* spores in RPMI 1640 medium.

Drugs	(MIC [µg/ml])	
	PCZ	ICZ
No Inhibitor	8 (32)	8 (32)
AA (1.5 nM)	8 (32)	16 (32)
BHAM (1.5 µM)	8 (32)	8 (32)
AA+BHAM(1.5 nM+1.5 µM)	0.25 (4)	0.25 (8)

Fluconazole had no effect on *R. oryzae* at all conditions tested.

MFC is given in parenthesis.

### Treatment with PCZ or ICZ in Combination with AA and BHAM Increases Intracellular ROS Accumulation and Superoxide Dismutase Activity

**Figure 1:**
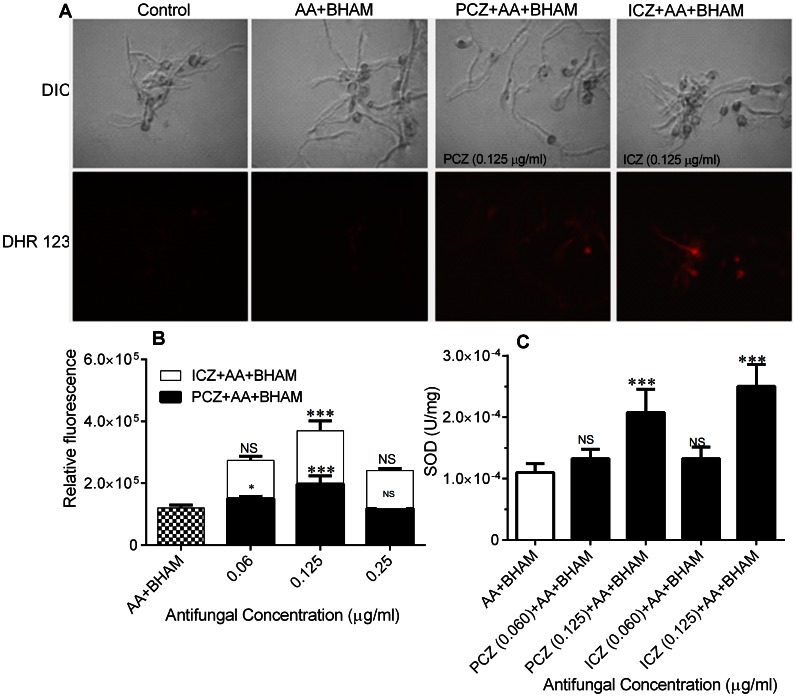
Intracellular ROS accumulation in *R.* *oryzae* germlings treated with PCZ or ICZ plus the mitochondrial inhibitors AA and BHAM, germlings treated with only AA and BHAM, and untreated control germlings stained with DHR-123 detected using fluorescence microscopy and a spectrophotometer. (A) Staining with DHR-123 showing increased red fluorescence. DIC, differential interference contrast. (B) Relative fluorescence of cells stained with DHR-123. The fluorescence was detected with excitation at 500 nm and emission at 550 nm. (C) SOD activity of treated and untreated control *R. oryzae* cells. P values for this and subsequent figures are denoted as follows: *, *p*<0.05, **, *p*<0.001, and ***, *p*<0.0001, while NS, *p*>0.05, compared to AA+BHAM treated controls. The experiments were performed in triplicate and repeated three times. Error bars indicate standard deviations for this and subsequent figures.

ROS and mitochondria play important role in apoptosis induction under both physiological and pathological conditions. Interestingly, mitochondria are both source and target of ROS [27]. We observed that *R. oryzae* germlings treated with PCZ or ICZ in combination with AA and BHAM, exhibited increased intracellular ROS production (3.5 fold) than did untreated germlings and those treated with AA and BHAM alone (Figure 1A and B). At a higher concentration of PCZ/ICZ (0.25 µg/ml) in combination with AA+BHAM a decrease in fluorescence was observed, probably due to faster cell death rate.

Likewise, *R. oryzae* germlings treated with PCZ and ICZ alone at much higher concentrations (8 µg/ml) had higher ROS levels, compared to untreated controls (Figure S1). At a lower concentration of PCZ/ICZ (<0.06 µg/ml), their combination with AA+BHAM resulted in no difference in ROS generation, compared to untreated or AA+BHAM treated controls (data not shown).

Scavenging of excess ROS is accomplished by the antioxidant enzymes catalase, glutathione peroxidase and antioxidants such as glutathione and vitamins C and E, for protection against oxidative damage. In contrast, superoxide is eliminated by the enzyme SOD [23]. We observed an increase in SOD activity and intracellular ROS levels in *R. oryzae* germlings. Also, we found that the SOD activity was 6.4 fold higher in *R. oryzae* germlings treated with AA+BHAM and PCZ (0.125 µg/ml) and 5-fold higher when treated with AA+BHAM and ICZ (0.125 µg/ml), than in germlings treated with AA and BHAM and in untreated germlings (Figure 1C).

### PCZ or ICZ in Combination with AA and BHAM Trigger a Decrease in Mitochondrial Membrane Potential and Increases Plasma Membrane Depolarization in *R. oryzae* Germlings

**Figure 2:**
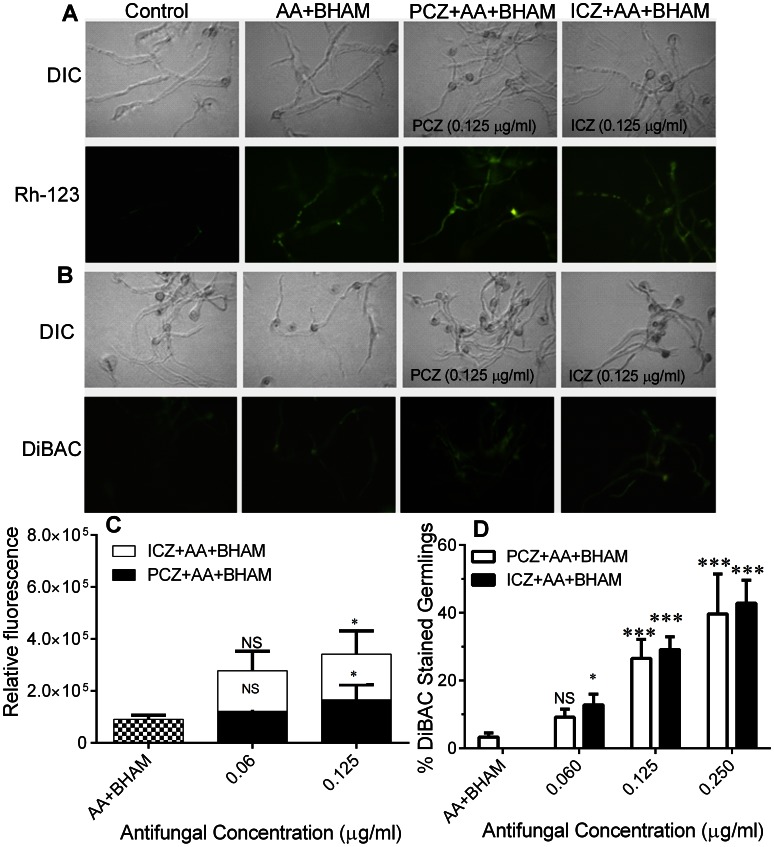
Changes in ΔΨ_m_ and plasma membrane depolarization in *R.* *oryzae* germlings triggered by treatment with PCZ or ICZ combined with AA and BHAM. (A) Stains of germlings with Rh-123 showing increased green fluorescence, indicating loss of ΔΨ_m_. (B) Staining of germlings with DiBAC showing increased green fluorescence, indicating loss of viability owing to increased membrane permeability. DIC, differential interference contrast. (C) Relative fluorescence of germlings stained with Rh-123. (D) Percentages of *R. oryzae* germlings stained with DiBAC. *, *p*<0.05, ***, *p*<0.0001, while NS, *p*>0.05, compared to AA+BHAM treated controls.

As shown in Figure 2 A and B treatment with PCZ and ICZ alone at higher concentrations (8 µg/ml) and in combination with AA and BHAM (at much lower concentrations, 0.125 µg/ml) reduced the mitochondrial membrane potential (ΔΨ_m_) and increased plasma membrane depolarization of *R. oryzae* germlings as evidenced by an increase in the number and intensity of fluorescent germlings over those of untreated germlings and germlings treated with AA and BHAM. The mean fluorescence was 3-fold higher in *R. oryzae* germlings treated with PCZ (0.125 µg/ml) and ICZ (0.125 µg/ml) in combination with AA and BHAM, than control germlings (Figure 2 C and S1). The decreased mitochondrial membrane potential was restored in the presence of the ROS scavenger NAC (data not shown).

Apoptosis affects the ionic gradient across the plasma membrane, leading to ionic imbalance and depolarization of the plasma membrane [12]. Therefore, to further elucidate the mechanism of the fungicidal action of PCZ and ICZ in the presence of both AA and BHAM, we used the plasma membrane potential-sensitive DiBAC which enters depolarized cells [13]. Untreated *R. oryzae* germlings and germlings treated with AA and BHAM were used as controls. We observed increased uptake of DiBAC stain in *R. oryzae* germlings treated with PCZ or ICZ, in combination with AA and BHAM, which was consistent with enhanced hyphal membrane damage and increased cellular permeability (Figure 2 B and D). These results suggested that PCZ and ICZ trigger depolarization of the plasma membrane and activate the apoptotic signaling machinery via cation overload or anion efflux.

### PCZ or ICZ in Combination with AA and BHAM Increases Cytochrome *c* Release from Mitochondria in *R. oryzae* Germlings

**Figure 3:**
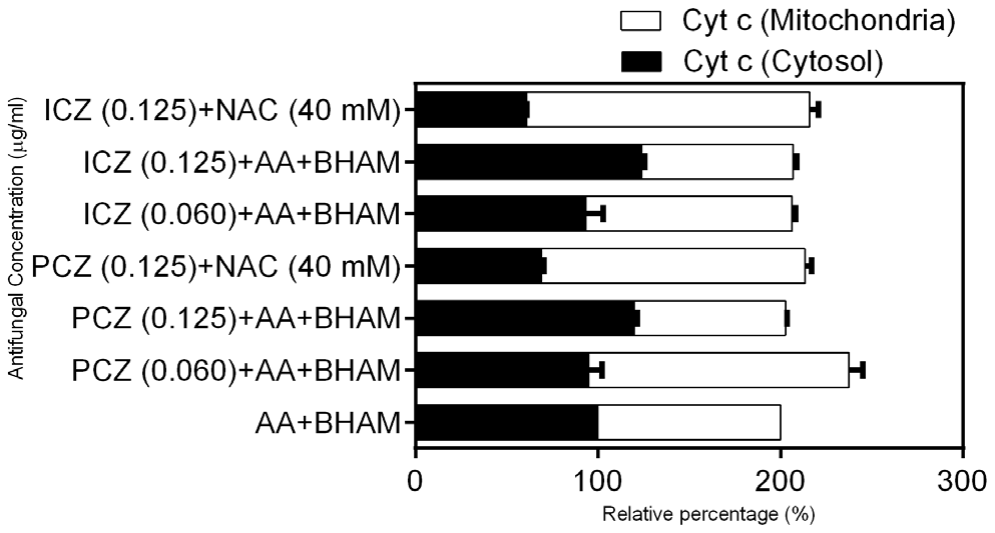
Effect of treatment with PCZ or ICZ combined with AA and BHAM on cyt *c* release from mitochondria in *R.* *oryzae* cells. Treatment with PCZ or ICZ in combination with AA and BHAM produced lower cyt *c* levels in mitochondria than did treatment with AA and BHAM alone. Release of cyt *c* to the cytosol was assessed by measuring the absorbance at 550 nm with a Beckman DU-640 spectrophotometer.

Translocation of mitochondrial cyt *c* to the cytosol is a critical event in apoptosis [12]. We found that the levels of cyt *c* in mitochondria and the cytosol in *R. oryzae* germlings treated with PCZ or ICZ in conjunction with AA and BHAM -induced apoptosis differed from those in untreated and AA and BHAM treated germlings. Specifically, the relative percentage of cyt *c* in *R. oryzae* mitochondria decreased by 1.5-fold (PCZ, 0.6–0.125 µg/ml) and 1.7-fold (ICZ, 0.6–0.125 µg/ml) in combination with AA and BHAM compared to untreated and AA and BHAM treated *R. oryzae* germlings. However, the cyt *c* levels in the cytosol were higher than in the mitochondria, indicating that treatment with PCZ or ICZ (0.6–0.125 µg/ml), in combination with AA and BHAM induced the release of cyt *c* from mitochondria in *R. oryzae* germlings (Figure 3).

### ROS Production, ΔΨ_m_ and Plasma Membrane Depolarization are Reversed by the NAC in *R. oryzae* Germlings Treated with PCZ or ICZ in Combination with AA and BHAM

As shown in Figure 2A and S1, generation of ROS in *R. oryzae* germlings treated with PCZ or ICZ alone and in combination with AA and BHAM was much greater than that in untreated germlings. In the presence of NAC, ROS production and membrane depolarization decreased leading to increased survival of the *R. oryzae* germlings treated with AA and BHAM in combination with PCZ or ICZ (data not shown). This indicated that intracellular ROS accumulation is a major contributor in *R. oryzae* germlings mortality, induced by treatment with AA and BHAM, in combination with triazoles. A decrease in cyt *c* relase from mitochondria to cytosol was observed in cells treated with PCZ/ICZ+AA and BHAM in the presence of NAC (Figure 3). Similarly, when germlings were exposed to PCZ/ICZ alone in the presence of NAC, ROS production and ΔΨ_m_ were decreased compared to untreated control (Figure S1).

### PCZ or ICZ in Combination with AA and BHAM Activates Metacaspases (Caspase- like Activity) in *R. oryzae* Germlings

**Figure 4:**
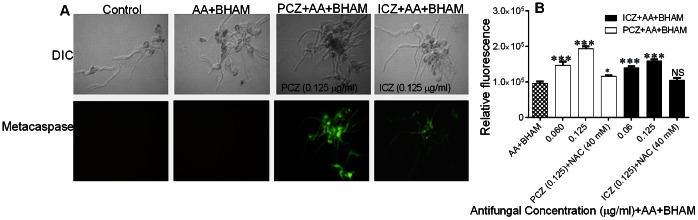
Effect of treatment with PCZ or ICZ in combination with AA and BHAM on caspase-like activity in *R.* *oryzae* germlings as determined using FITC-VAD-FMK probe. (A) Caspase-like activity as visualized under a fluorescence microscope. DIC, differential interference contrast. (B) Relative fluorescence of germlings stained with FITC-VAD-FMK. Untreated control cells did not exhibit any fluorescence. ***, *p*<0.0001, NS, p>0.05, compared to AA+BHAM treated controls.

Caspases are activated in the early stages of apoptosis and play a central role in the apoptotic cascade [20], [28]. We detected caspase-like activity in *R. oryzae* germlings treated with PCZ or ICZ (0.125 µg/ml) in combination with AA and BHAM (Figure 4 A and B). In contrast, no caspase-like activity was detected in untreated or AA and BHAM treated control germlings. *R. oryzae* germlings pretreated with the ROS scavenger NAC failed to show caspase-like activity.

### PCZ or ICZ in Combination with AA and BHAM Induces Morphological Changes Indicative of Apoptosis in *R. oryzae* Germlings

**Figure 5:**
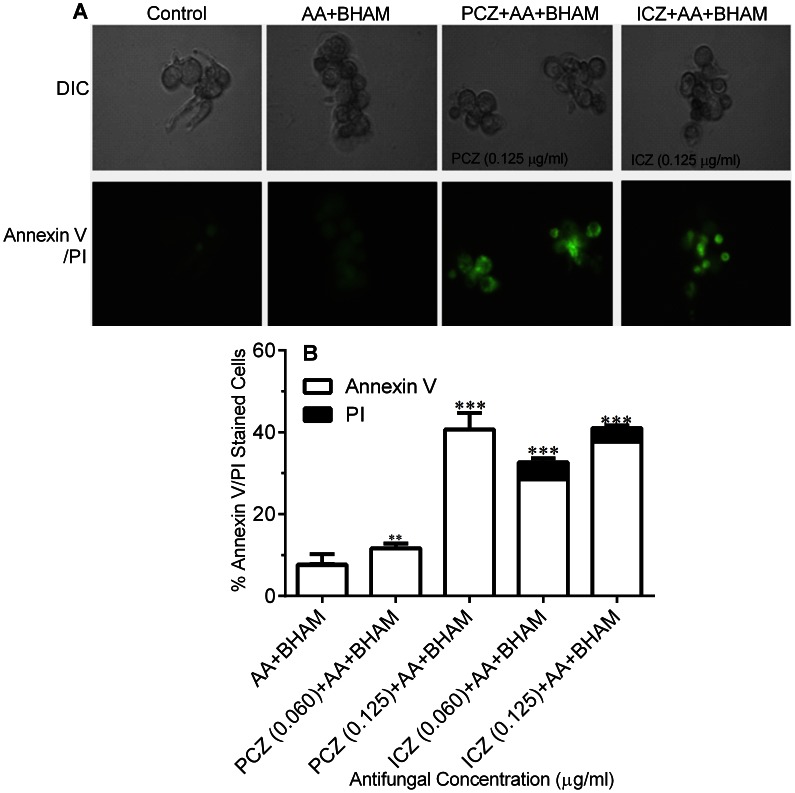
Representative fluorescent images of *R.* *oryzae* cells treated with PCZ or ICZ in combination with AA and BHAM, cells treated with only AA and BHAM, and untreated control cells. (A) Annexin V/PI double staining showing apoptotic cells (green fluorescence) and necrotic cells (red fluorescence). DIC, differential interference contrast. (B) Percentages of cells displaying annexin V/PI double staining. **, *p*<0.001, and ***, *p*<0.0001, compared to AA+BHAM treated controls.

Our observation of mitochondrial injury and ROS accumulation prompted us to further examine the occurrence of apoptosis under these conditions. To that end, we examined the extent of PS externalization in *R. oryzae* cells using annexin V- FITC and PI staining to check plasma membrane integrity. Cells exposed to PCZ or ICZ in combination with AA and BHAM were stained green, indicating the presence of PS on the outer surface of the plasma membrane (Figure 5A). The number of annexin V -FITC positive cells were 45±2% and 40±5% among cells treated with PCZ and ICZ, respectively, along with AA and BHAM, whereas only 30±5%–50±5% cells treated with PCZ or ICZ alone were positive for annexin V-FITC (Figure S2). Also, untreated and AA and BHAM treated control cells exhibited no detectable fluorescence (Figure 5 B). In the presence of NAC, few cells (5–7%) treated with PCZ or ICZ in combination with AA and BHAM exhibited PS externalization (data not shown).

**Figure 6:**
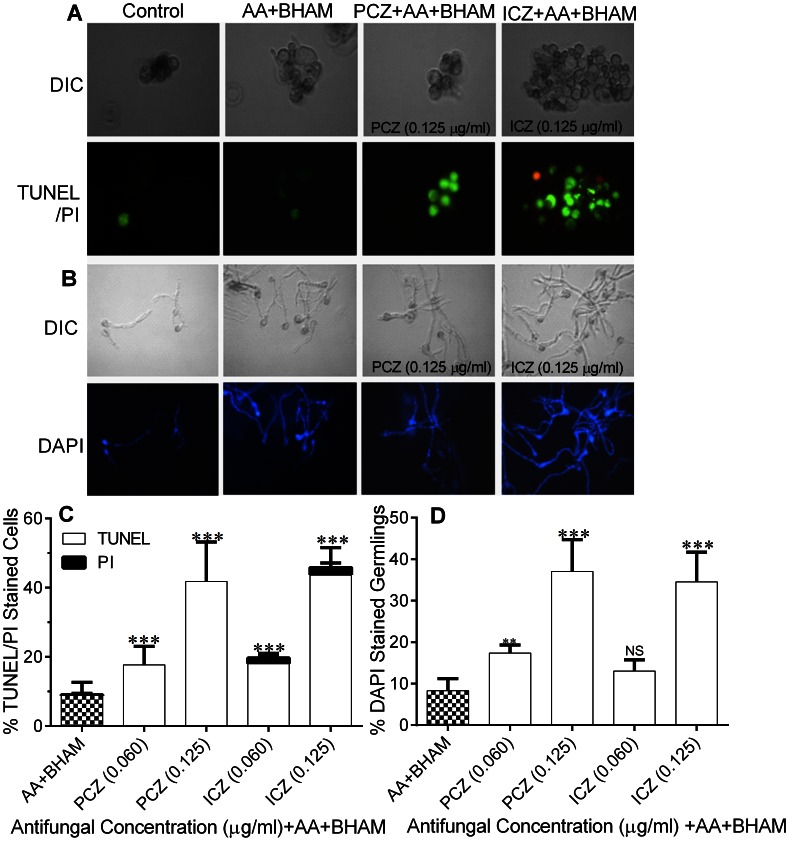
DNA and nuclear fragmentation in *R.* *oryzae* germlings treated with PCZ or ICZ in combination with AA and BHAM, germlings treated with only AA and BHAM, and untreated control germlings visualized using fluorescence microscopy. (A) TUNEL/PI staining DNA fragmentation of *R. oryzae* germlings. (B) DAPI staining of nuclear condensation of *R. oryzae* germlings. DIC, differential interference contrast. (C and D) Percentages of *R. oryzae* germlings displaying loss of membrane integrity (PI+), DNA strand breaks (TUNEL+), and chromatin condensation (DAPI+). **, *p*<0.001, and ***, *p*<0.0001, while NS, *p*>0.05, compared to AA+BHAM treated controls.

Similarly, we found that *R. oryzae* cells treated with PCZ or ICZ in combination with AA and BHAM had a TUNEL positive phenotype and chromatin condensation (Figure 6 A–D), indicating DNA fragmentation and margination. In contrast, none of the untreated cells had nuclear staining for TUNEL and 5±1%–10±1% of AA and BHAM exhibited this staining (Figure 6 A and B). Finally, we assessed nuclear fragmentation in *R. oryzae* germlings treated with PCZ or ICZ in combination with AA and BHAM using the nucleic acid probe DAPI [20]. In control germlings, chromatin appeared as a single round spot with a normal appearance (Figure 6B). In contrast, germlings exposed to PCZ or ICZ plus AA and BHAM exhibited distribution of chromatin fragments in the cells (Figure 6 B and D). The addition of NAC to treatment with PCZ or ICZ in combination with AA and BHAM reduced the percentage of germlings with nuclear fragmentation (10%±1% to 15%±1%).

## Discussion

In our study, we demonstrated for the first time that simultaneous inhibition of classical and alternative respiratory pathways in combination with PCZ or ICZ increases the sensitivity of *R. oryzae* to triazoles and this activity was mediated through induction of apoptosis. The mitochondrial respiratory chains in humans and in fungal pathogens are fundamentally different [29]; hence our findings may lead to therapeutic strategies that enhance the activity of existing antifungal drugs for Mucorales control. Specifically, we found that incubation of *R. oryzae* germlings with PCZ or ICZ along with AA and BHAM greatly reduced the MICs of PCZ and ICZ and increased ROS accumulation, cell membrane disruption, mitochondrial membrane hyperpolarization, apoptosis, and activation of caspase-like activity. Our in vitro susceptibility data are in agreement with the results of prior studies demonstrating that inhibition of the respiratory networks accounted for increased susceptibility of *Candida parapsilosis* to caspofungin and of *Aspergillus flavus* and *Aspergillus fumigatus* to phenolics [10], [30]. Also, Martinez et al. [9] studied *Rhizopus stolonifer* mitochondria using specific substrates and inhibitors of mitochondrial enzymes and showed that it contains an alternative oxidase and glycerol phosphate shuttle in addition to complexes I, II, III, IV, and V.

The most important mitochondrila function is generation of ATP via oxidative phosphorylation, in which ΔΨ_m_ plays an essential role in the regulation of the electrochemical gradient through the electron transport chain [18], [31]. Abrogation of electron transfer results in profound perturbations of oxidative phosphorylation which can enhance ROS generation, ΔΨ_m_ [18], [32], [33]. Studies of *Saccharomyces cerevisiae* have implicated a role for mitochondria in induction of loss of azole effectiveness [34], [35].

In the present study, the apoptosis-induction mechanism and fungicidal activities of AA and BHAM in combination with PCZ or ICZ in *R. oryzae* germlings correlated with increased accumulation of ROS (Figure 1 and Table 1). Cells have effective detoxification mechanisms, including enzymes such as catalase, SOD, and glutathione peroxidase and antioxidants such as glutathione and vitamins C and E, for protection against oxidative damage [23]. Authors have reported that such protective mechanisms are activated in *A*. *fumigatus*, *Candida albicans*, *S. cerevisiae*, and *Schizosaccharomyces pombe* cells, in response to treatment with amphotericin B and mutations affecting replication initiation [23], [36]. The increased SOD activity we observed in *R. oryzae* cells treated with PCZ or ICZ in combination with AA and BHAM was consistent with reports of increased SOD activity in cells of *Neurospora crassa*, *Fusarium decemcellulare*, and other filamentous fungi in response to treatment with antifungal drugs [23], [36]. Furthermore, addition of the antioxidant NAC not only prevented ROS generation but also attenuated the antifungal effect of PCZ and ICZ in combination with AA and BHAM, indicating that ROS are an important mediator of the antifungal action of triazoles.

Authors have described the role of mitochondria in apoptotic death of *C. albicans*, *A. fumigatus*, and *A. flavus* cells in the presence of various agents, such as plagiochin E, psacotheasin, farnesol, and dill oil [18], [37], [38]. Similarly, we found that PCZ and ICZ in combination with AA and BHAM significantly decreased the ΔΨ_m_ in a dose-dependent manner in *R. oryzae* cells, suggesting mitochondrial dysfunction (Figure 2 A and B). These data suggested that PCZ and ICZ induced cell membrane depolarization and mitochondrial membrane hyperpolarization in *R. oryzae* germlings.

Translocation of mitochondrial cyt *c* to the cytosol is also a critical event in apoptosis. In mammalian cells, after the release of cyt *c* into the cytosol, cyt *c* binds to apoptotic protease-activating factor to form a complex with caspase-9, resulting in activation of the caspase cascade and thus inducing apoptosis [20], [39]. Release of cyt *c* requires an increase in mitochondrial membrane permeability during apoptosis [20]. Our results in the present study are consistent with those of that linked ROS formation, changes in ΔΨ_m_, and cyt *c* release with apoptosis [37], [40]. Our study indicates that ROS and release of cyt *c* into the cytosol of germlings treated with PCZ or ICZ in combination with AA and BHAM induce the activity of enzymes with mammalian caspase-like proteolytic activity. However, the exact source of such enzymatic activity, remains undefined in fungi (*R. oryzae*). In contrast, cells treated with NAC did not exhibit any caspase-like activity. Thus, our findings supports that inhibiting respiratory pathways along with ergosterol biosynthesis by triazoles and other antifungal drugs deserves further study, as it may be a useful adjunct therapeutic strategy for these refractory Mucorales infections.

Determining the in vivo significance of these findings along with specificity and toxicity issues for their effect on the mitochondrial pathway of humans requires further investigation. The toxicity issues can be resolved by identifying differences in mitochondrial respiratory pathways in fungi and humans using in silico studies, designing fungus-specific mitochondrial inhibitors, and identifying other strategies for targeting respiratory pathway in Mucorales spp. and molds.

## Supporting Information

Figure S1 **Changes in ROS levels and ΔΨ_m_ in** ***R. oryzae*** **germlings triggered by treatment with PCZ or ICZ alone and in the presence or absence of NAC.** (A) DHR-123 staining of germlings showing increased red fluorescence. (B) Rh-123 staining of germlings showing increased green fluorescence, indicating loss of ΔΨ_m_. DIC, differential interference contrast. (C) Relative fluorescence of *R. oryzae* cells stained with DHR-123. (D) Relative fluorescence of *R. oryzae* cells stained with Rh-123. The experiments were performed in triplicate and repeated three times. Error bars indicate standard deviations. ***,*p*<0.0001, NS, *p*>0.05, compared to AA+BHAM untreated controls.
(TIF)

Figure S2 **Representative fluorescent images of** ***R. oryzae*** **cells treated with PCZ or ICZ and untreated control cells.** (A) Annexin V/PI double staining showing apoptotic cells (green fluorescence) and necrotic cells (red fluorescence). DIC, differential interference contrast. (B) Percentages of cells displaying annexin V/PI double staining. ***, *p*<0.0001, compared to AA+BHAM untreated controls.
(TIF)
