# Supplementary figures and images for: Mitochondrial Respiratory Pathways Inhibition in Rhizopus oryzae Potentiates Activity of Posaconazole and Itraconazole via Apoptosis
Source: PLoS One. 2013 May 17;8(5):e63393. doi: 10.1371/journal.pone.0063393 (PMC3656966; doi:10.1371/journal.pone.0063393)

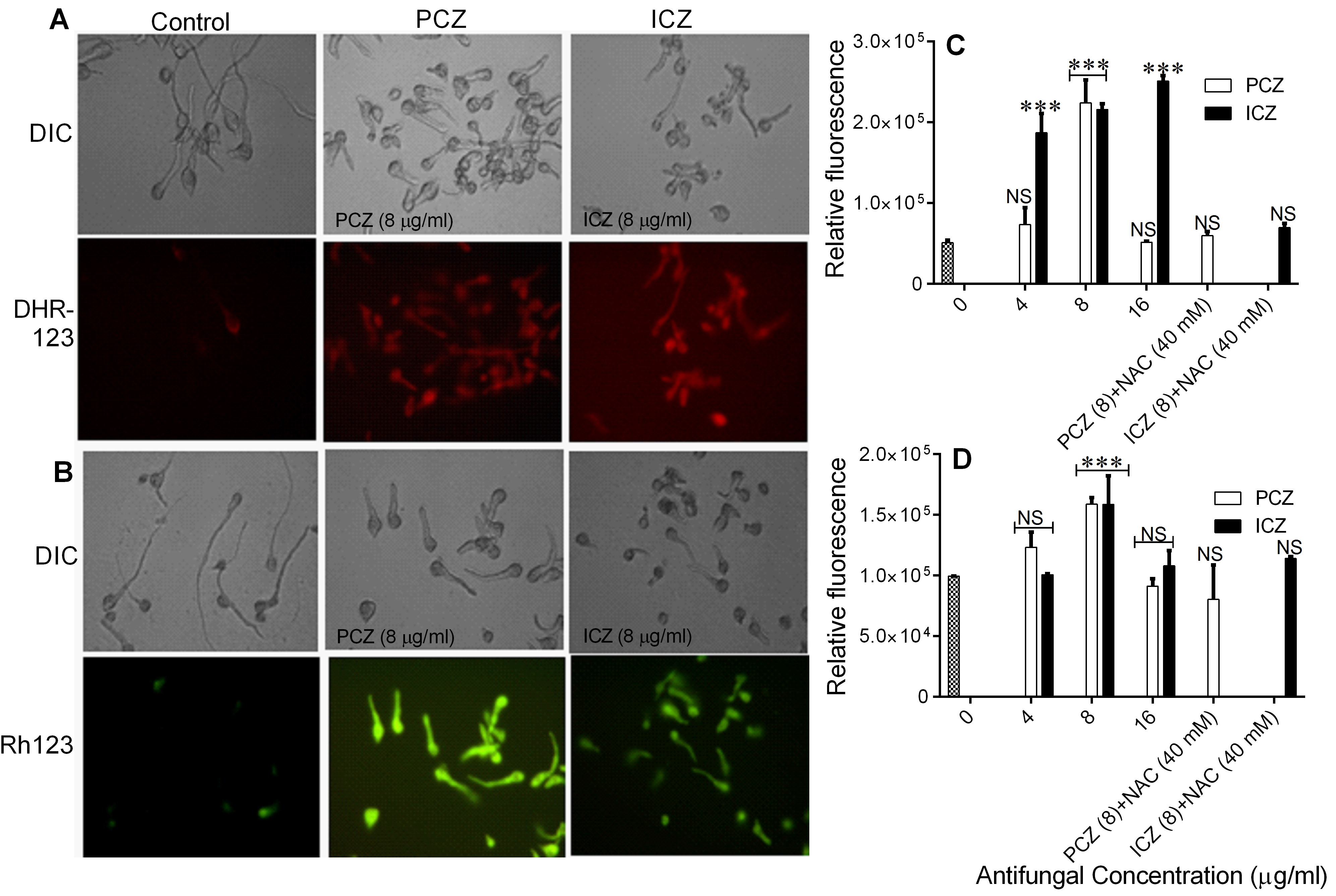

Supplement: Figure S1 — Changes in ROS levels and ΔΨm in R. oryzae germlings triggered by treatment with PCZ or ICZ alone and in the presence or absence of NAC. (A) DHR-123 staining of germlings showing increased red fluorescence. (B) Rh-123 staining of germlings showing increased green fluorescence, indicating loss of ΔΨm. DIC, differential interference contrast. (C) Relative fluorescence of R. oryzae cells stained with DHR-123. (D) Relative fluorescence of R. oryzae cells stained with Rh-123. The experiments were performed in triplicate and repeated three times. Error bars indicate standard deviations. ***,p<0.0001, NS, p>0.05, compared to AA+BHAM untreated controls. (TIF) [file pone.0063393.s001.tif]

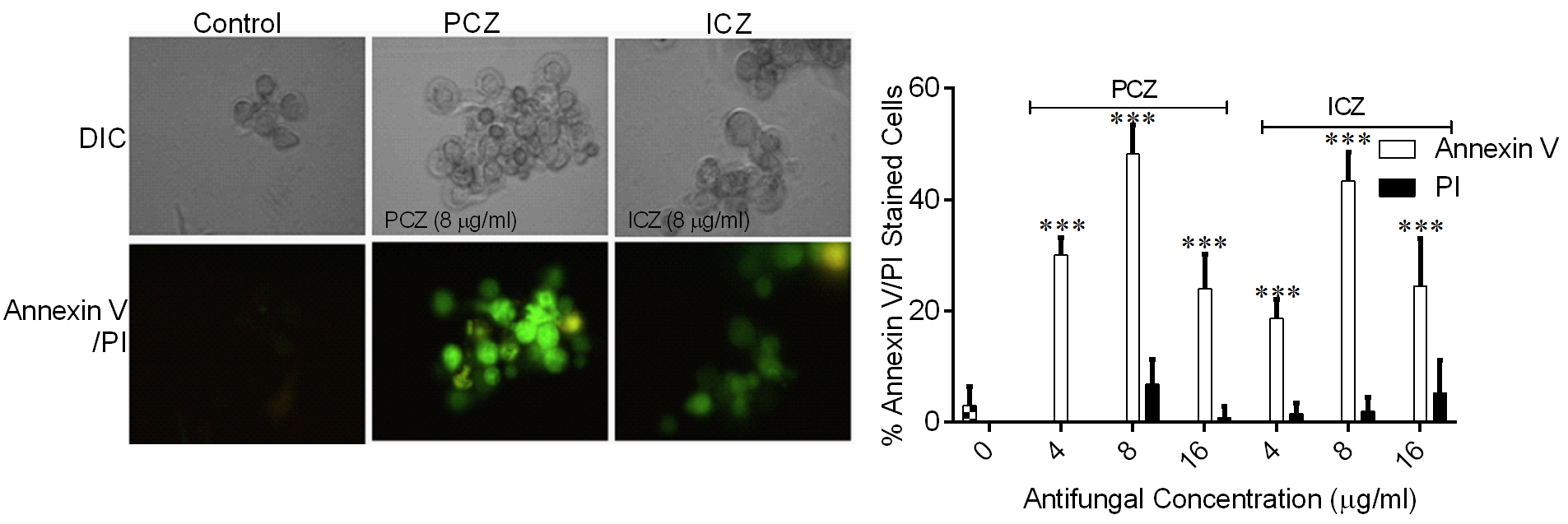

Supplement: Figure S2 — Representative fluorescent images of R. oryzae cells treated with PCZ or ICZ and untreated control cells. (A) Annexin V/PI double staining showing apoptotic cells (green fluorescence) and necrotic cells (red fluorescence). DIC, differential interference contrast. (B) Percentages of cells displaying annexin V/PI double staining. ***, p<0.0001, compared to AA+BHAM untreated controls. (TIF) [file pone.0063393.s002.tif]
